# CCDC189 affects sperm flagellum formation by interacting with CABCOCO1

**DOI:** 10.1093/nsr/nwad181

**Published:** 2023-06-26

**Authors:** Mengyue Wang, Junyan Kang, Zhiming Shen, Yingchun Hu, Min Chen, Xiuhong Cui, Hongbin Liu, Fei Gao

**Affiliations:** State Key Laboratory of Stem Cell and Reproductive Biology, Institute of Zoology, Chinese Academy of Sciences, Beijing 100020, China; Institute for Stem Cell and Regeneration, Chinese Academy of Sciences, Beijing 100020, China; University of Chinese Academy of Sciences, Beijing 101499, China; Department of Ophthalmology, Shanghai Ninth People's Hospital, Shanghai Jiao Tong University School of Medicine, Shanghai Key Laboratory of Orbital Diseases and Ocular Oncology, Shanghai 200031, China; State Key Laboratory of Stem Cell and Reproductive Biology, Institute of Zoology, Chinese Academy of Sciences, Beijing 100020, China; Institute for Stem Cell and Regeneration, Chinese Academy of Sciences, Beijing 100020, China; University of Chinese Academy of Sciences, Beijing 101499, China; Core Facilities, College of Life Sciences, Peking University, Beijing 100871, China; State Key Laboratory of Stem Cell and Reproductive Biology, Institute of Zoology, Chinese Academy of Sciences, Beijing 100020, China; Institute for Stem Cell and Regeneration, Chinese Academy of Sciences, Beijing 100020, China; State Key Laboratory of Stem Cell and Reproductive Biology, Institute of Zoology, Chinese Academy of Sciences, Beijing 100020, China; Institute for Stem Cell and Regeneration, Chinese Academy of Sciences, Beijing 100020, China; Beijing Institute for Stem Cell and Regenerative Medicine, Beijing 100020, China; Center for Reproductive Medicine, Shandong University, Jinan 250100, China; State Key Laboratory of Stem Cell and Reproductive Biology, Institute of Zoology, Chinese Academy of Sciences, Beijing 100020, China; Institute for Stem Cell and Regeneration, Chinese Academy of Sciences, Beijing 100020, China; Beijing Institute for Stem Cell and Regenerative Medicine, Beijing 100020, China; University of Chinese Academy of Sciences, Beijing 101499, China

**Keywords:** MMAF, *Ccdc189*, *Cabcoco1*, flagellum formation, radial spoke

## Abstract

Multiple morphological abnormalities of the sperm flagella (MMAF) are one of the major causes of male infertility and are characterized by multiple defects. In this study, we found that the coiled-coil domain-containing 189 (*Ccdc189*) gene was predominantly expressed in mouse testes and that inactivation of the *Ccdc189* gene caused male infertility. Histological studies revealed that most sperm from *Ccdc189-deficient* mice carried coiled, curved or short flagella, which are typical MMAF phenotypes. Immunoelectron microscopy showed that the CCDC189 protein was located at the radial spoke of the first peripheral microtubule doublet in the sperm axoneme. A CCDC189-interacting protein, CABCOCO1 (ciliary-associated calcium-binding coiled-coil protein 1), was discovered via co-immunoprecipitation and mass spectrometry, and inactivation of *Cabcoco1* caused malformation of sperm flagella, which was consistent with findings obtained with *Ccdc189-deficient* mice. Further studies revealed that inactivation of CCDC189 caused downregulation of CABCOCO1 protein expression and that both CCDC189 and CABCOCO1 interacted with the radial-spoke-specific protein RSPH1 and intraflagellar transport proteins. This study demonstrated that *Ccdc189* is a radial-spoke-associated protein and is involved in sperm flagellum formation through its interactions with CABCOCO1 and intraflagellar transport proteins.

## Teaser

Discovering a novel candidate gene for MMAF disease and unraveling the roles of CCDC189 and CABCOCO1 in sperm flagellar development.

## INTRODUCTION

Infertility is a multifactorial pathological condition affecting ∼10%–15% of couples worldwide, and nearly half of these infertility cases involve male infertility [1]. Male infertility often manifests as decreased sperm count (oligozoospermia), reduced sperm count (asthenozoospermia), a high proportion of morphologically defective sperm (teratozoospermia) or a combination of these abnormalities [2–4]. An estimated 30%–50% of male infertility is caused by genetic alterations. Therefore, considerable effort has been made to identify and characterize the genes required for male germ cell development [5].

Spermatogenesis is a complex developmental process that typically consists of three stages: mitosis of spermatogonia stem cells, meiosis of spermatocytes, and spermiogenesis. Spermiogenesis is the final stage of spermatogenesis, where round spermatids terminally differentiate into spermatozoa. This process includes acrosome and flagellum formation, nuclear condensation and cytoplasmic exclusion [6,7]. Impairment of any of these processes causes defects in sperm morphogenesis. The flagella of normal sperm are composed of nine peripheral microtubule doublets (MTDs) surrounding a pair of singlet microtubules called the central pair (CP). The peripheral doublets are connected to each other by nexin-dynein regulatory complexes (NDRCs) and to the central pair by radial spokes (RSs) [8].

Multiple morphological abnormalities of the sperm flagella (MMAF) constitute one of the most severe forms of sperm defects critical for male infertility and are characterized by numerous non-motile spermatozoa in ejaculated semen that exhibit flagellar abnormalities, including short, coiled, absent and irregular flagella [9]. MMAF is caused mainly by genetic mutations, and ∼40 MMAF-associated genes have been reported in the past 10 years. The main pathogenic gene families include the DNAH protein family [10–16], coiled-coil domain-containing (CCDC) protein family [17], cilia- and flagella-associated protein (CFAP) family and other protein-encoding genes associated with flagellum biogenesis and morphogenesis [5,9,18–27]. Mutations or deletions of these genes usually cause MMAF in human patients and animal models. Although MMAF is not a rare autosomal-recessive inherited disorder in humans, the genetic etiology of a large proportion of MMAF cases remains elusive [2,28], and the recently identified MMAF-associated genes explain only 30%–60% of the MMAF cases in different cohorts [9]. Therefore, it remains necessary to improve the ability to distinguish single candidate genes in human MMAF samples, and this study may provide information to guide ICSI (intracytoplasmic sperm injection) treatment for MMAF patients.

CCDC proteins are involved in a wide range of physiological and pathological processes. An increasing number of CCDC proteins have been shown to be involved in flagellar morphogenesis. The genes in this family, such as *Ccdc9, Ccdc11, Ccdc33, Ccdc39, Ccdc42, Ccdc47, Ccdc63* and *Ccdc172*, are also involved in spermatogenesis [17,29–32]. Mutations in these genes cause defects in sperm flagellum biogenesis and male infertility. It has been reported that the CCDC189 protein is located in the flagellum of rat sperm [33], and mutation of the *Ccdc189* gene is associated with male infertility in Nordic Red dairy cattle [34]. These results suggest that *Ccdc189* is probably involved in the biogenesis of sperm flagellum, however, the physiological functions of this gene have not been investigated to date.

To investigate the function of *Ccdc189* in sperm development, a *Ccdc189*-knockout mouse model was generated in this study. We found that *Ccdc189^−/−^*males were infertile. The morphology of the sperm was abnormal as they presented with coiled, curved or short flagella. Further investigations revealed that the CCDC189 protein was located at the radial spoke of the first microtubule doublet and interacted with ciliary-associated calcium-binding coiled-coil protein 1 (CABCOCO1). We also found that the knockout of *Cabcoco1* caused defects in sperm flagellum formation, which was consistent with the findings with *Ccdc189^−/−^*mice. This study demonstrated that *Ccdc189* is a radial-spoke-associated protein and plays important roles in sperm flagellum formation by interacting with CABCOCO1.

## RESULTS

### Ccdc189-knockout male mice are sterile

**Figure 1.**
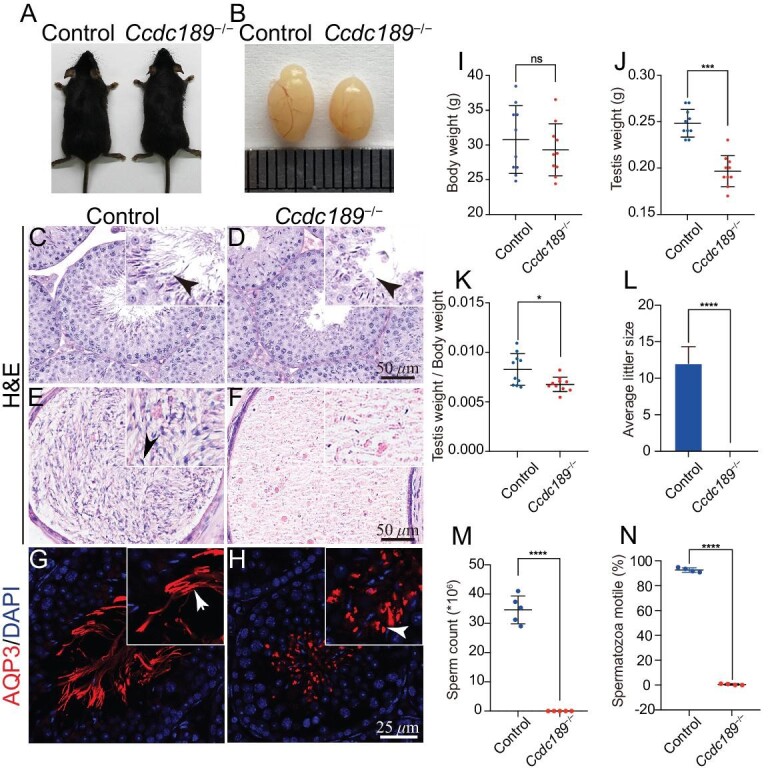
Inactivation of *Ccdc189* causes male infertility. (A) No developmental abnormalities were observed in adult *Ccdc189*^−/−^ mice, and the body weight was comparable to that of control mice (I). (B and J) The size of testes from *Ccdc189*^−/−^ mice was smaller than that of control mice. (K) The ratio of testis/body weight was significantly decreased in *Ccdc189*^−/−^ mice. (L) Fertility test of control and male *Ccdc189*^−/−^ mice. The histology of the seminiferous tubules in (C) control and (D) *Ccdc189*^−/−^ mice was examined by H&E staining. A large number of mature sperm were observed in the epididymis of (E) control mice, and a small number of mature sperm were noted in (F) *Ccdc189*^−/−^ mice. Sperm tails in the testes of (G) control mice were labeled with AQP3 (red), and scattered AQP3 signal was detected in the testes of (H) *Ccdc189*^−/−^ mice. (M) The number of sperm was dramatically reduced in *Ccdc189*^−/−^ mice. (N) The motility of sperm was dramatically reduced in *Ccdc189*^−/−^ mice compared to control mice.

The expression of *Ccdc189* in mice was measured by reverse transcription quantitative real-time PCR (RT−PCR) and western blotting. As shown in Supplementary Fig. 1A and B, the mRNA of *Ccdc189* was predominantly expressed in testes, and it was first detected in testes in mice that were 3 weeks old. CCDC189 protein was detected only in testes, first in 3-week-old mice, and the expression level was higher in adult mice (Supplementary Fig. 1C and D). To investigate the physiological functions of *Ccdc189* in mouse germ cell development, we generated a *Ccdc189^−/−^* mouse model using clustered regularly interspaced short palindromic repeats (CRISPR)−Cas9 technology. As shown in Supplementary Fig. 2A, a truncated mutation was generated by deleting exons from 4 to 8. Using genomic polymerase chain reaction (PCR), successful knockdown of *Ccdc189* was confirmed (Supplementary Fig. 2B). The results of western blotting showed that the CCDC189 protein was completely absent in the testes of the *Ccdc189^−/−^* mice (Supplementary Fig. 2C), indicating that the *Ccdc189* gene was inactivated in the *Ccdc189^−/−^* mice. Adult *Ccdc189^−/−^*mice were grossly normal, and no obvious developmental abnormalities were observed. The size of the testes from the *Ccdc189^−/−^* mice was significantly decreased compared to that in the control mice, and the testis/body weight ratio was also significantly reduced in the *Ccdc189^−/−^*mice (Fig. 1A, B and I–K). The results of a fertility test showed that *Ccdc189^−/−^* males were completely infertile (Fig. 1L).

To explore the reasons for sterility in the *Ccdc189^−/−^* mice, the histology of the testes and cauda epididymis in control and *Ccdc189^−/−^*mice was analyzed by hematoxylin-eosin (H&E) staining (Fig. 1C–F). No obvious histological change was detected in the seminiferous tubules of the *Ccdc189^−/−^*mice (Fig. 1C and D). Sox9-positive Sertoli cells lined the peripheral region of seminiferous tubules in both the control and *Ccdc189^−/−^*mice (Supplementary Fig. 3A–H). DDX4-positive germ cells at different developmental stages were observed in seminiferous tubules in both the control and *Ccdc189^−/−^*mice, and no evident difference was noted (Supplementary Fig. 3I–P). A large number of mature spermatozoa were observed in the epididymis of the control mice, whereas very few sperm heads were observed in the *Ccdc189^−/−^* mice (Fig. 1E and F). Immunofluorescence of the flagella marker AQP3 protein showed fiber-like sperm tail structures in the seminiferous tubule lumen of the control mice. In contrast, only a punctate AQP3 signal was detected in the *Ccdc189^−/−^* mice (Fig. 1G and H). The results of computer-assisted sperm analysis (CASA) showed that the number and motility of the sperm from the *Ccdc189^−/−^* mice were dramatically reduced compared to those in control mice (Fig. 1M and N). These results indicated that in the early stage of germ cell development, *Ccdc189^−/−^* mice were not affected and that the *Ccdc189* gene was most likely involved in sperm morphogenesis.

### Ccdc189 is essential for sperm flagellar formation

**Figure 2.**
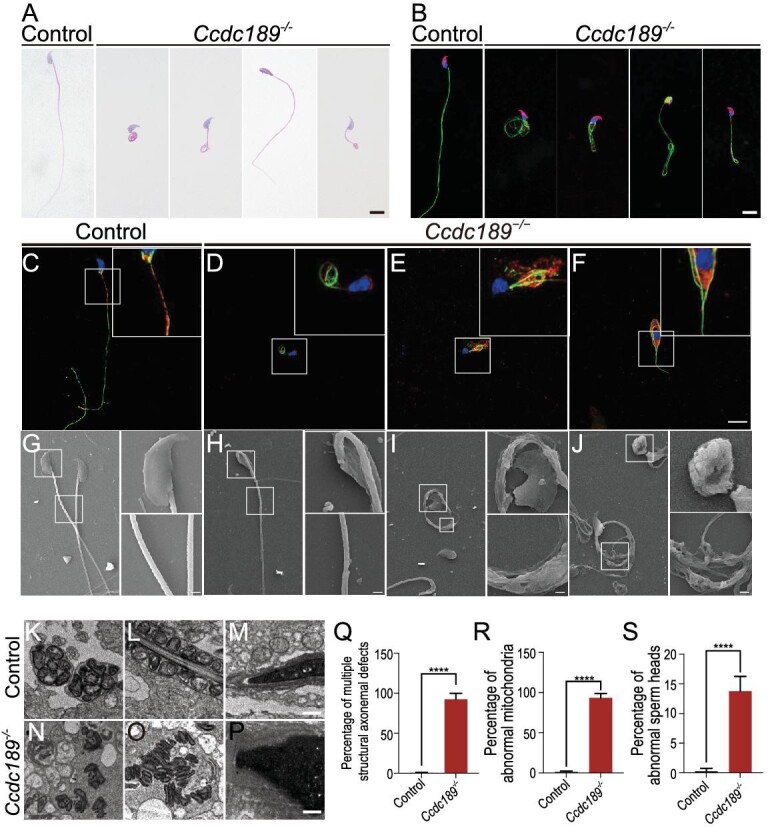
The morphology of sperm in *Ccdc189^−/−^* mice is abnormal. (A) The malformations of sperm flagella in *Ccdc189*^−/−^ mice. Sperm with short, coiled, helical flagella or abnormal heads were observed in *Ccdc189*^−/−^ mice. Scale bars = 10 μm. (B) The morphology of sperm was examined by immunofluorescence. Sperm tails were labeled with αTubulin (green), and acrosome was labeled with PNA (red). (C–F) Elongating spermatids were collected from *Ccdc189*^−/−^ and control testes and stained with IFT88 (red) and acetylated α-tubulin (green). Scale bars = 10 μm. (G–J) Sperm morphology was examined by scanning electron microscopy (SEM). (K–P) The morphology of sperm was examined by transmission electron microscopy (TEM). Scale bars = 1 μm. Quantitative analysis of sperm with a defect in the (Q) axoneme, (R) mitochondrial sheath and (S) sperm head in *Ccdc189*^−/−^ mice.

We further analyzed the process of spermiogenesis in *Ccdc189*-deficient mice by periodic-acid-Schiff (PAS) and hematoxylin staining. As shown in Supplementary Fig. 4A, stages 1–12 of the seminiferous epithelial cycle were observed in both the control and *Ccdc189^−/−^* mice, and no difference was noted between the control and *Ccdc189^−/−^* mice. Immunofluorescence staining for α-tubulin showed that the manchette in the *Ccdc189^−/−^* mice in Steps 11–12 was longer than that in the control mice. This implied that deletion of *Ccdc189* caused abnormal manchette formation. The abnormal manchette structure may have exerted an effect on flagellum biogenesis. Therefore, we further examined the morphology of mature sperm from control and *Ccdc189^−/−^* mice. As shown in Fig. 2A and B, sperm with normal morphology were observed in the control males. In contrast, most of the sperm in the *Ccdc189^−/−^* mice were malformed and presented with short, coiled flagella and abnormal heads, which was consistent with the sperm morphology observed in MMAF patients. The immunostaining results also showed that the morphology of α-tubulin-positive flagella was abnormal and that PNA-positive acrosomes were abnormal in the sperm from the *Ccdc189^−/−^* mice. We also examined the location of the IFT88 protein in control and *Ccdc189*-deficient sperm. As shown in Fig. 2C–F, IFT88 was located in the sperm flagella of control sperm (C) and co-localized with ace-tubulin during sperm elongation. In *Ccdc189*-deficient sperm (D–F), IFT88 protein was diffused in germ cells and not co-localized with ace-tubulin. To analyze the morphological defects in *Ccdc189*^−/−^ sperm, we performed an ultrastructural analysis of control and mutant sperm using scanning electron microscopy (SEM) and transmission electron microscopy (TEM). Crescent-shaped sperm heads with well-organized mitochondrial sheaths were observed in the control mice via SEM (Fig. 2G). Sperm with a round or missing head presented with a typical hook-shaped appearance (Fig. 2I and J), an impaired mitochondrial sheath (Fig. 2H) and a coiled fibrous sheath (Fig. 2I and J) in the *Ccdc189*^−/−^ mice. Well-organized sperm heads (Fig. 2M), well-organized mitochondrial sheaths (Fig. 2L), the normal 9 + 2 organization and NDRC structure (Fig. 2K) were observed in the control mice via TEM. The morphology of the head was normal in most sperm from the *Ccdc189^−/−^* mice (Fig. 2P), and a small number of sperm with abnormal heads were observed in the *Ccdc189^−/−^* mice (Fig. 2S). The mitochondrial sheath was disorganized in the *Ccdc189^−/−^* mice (Fig. 2O and R), and 9 + 2 structural abnormalities were consistently observed (Fig. 2N and Q). These results were consistent with the results of H&E staining and immunostaining.

To explore whether *Ccdc189*^−/−^ mice present with symptoms in addition to infertility, we examined airway epithelial cells in the tracheas of *Ccdc189*^−/−^ mice by H&E staining and immunohistochemistry (Supplementary Fig. 5A and B). The number of ciliated cells and the cilial morphology were not significantly changed in the *Ccdc189*^−/−^ mice compared to the control mice. Then, we further analyzed serum starvation induced primary cilial formation using mouse embryonic fibroblast cells (MEFs) from control and *Ccdc189*^−/−^ mice. As shown in Supplementary Fig. 5C, cilia were stained with an anti-acetylated tubulin antibody, a marker of primary cilia. We found no obvious differences in the number of primary cilia in the *Ccdc189*^−/−^ mouse fibroblast cells compared with the control cells (Supplementary Fig. 5D). We subsequently determined the cilial length of the *Ccdc189*^−/−^ mouse fibroblast cells and control cells, and no significant difference was detected in the length of *Ccdc189*^−/−^ mouse cilia compared with the control mouse cilia (Supplementary Fig. 5E).

### The CCDC189 protein localizes to the radial spoke of the first microtubule doublet of sperm flagella

**Figure 3.**
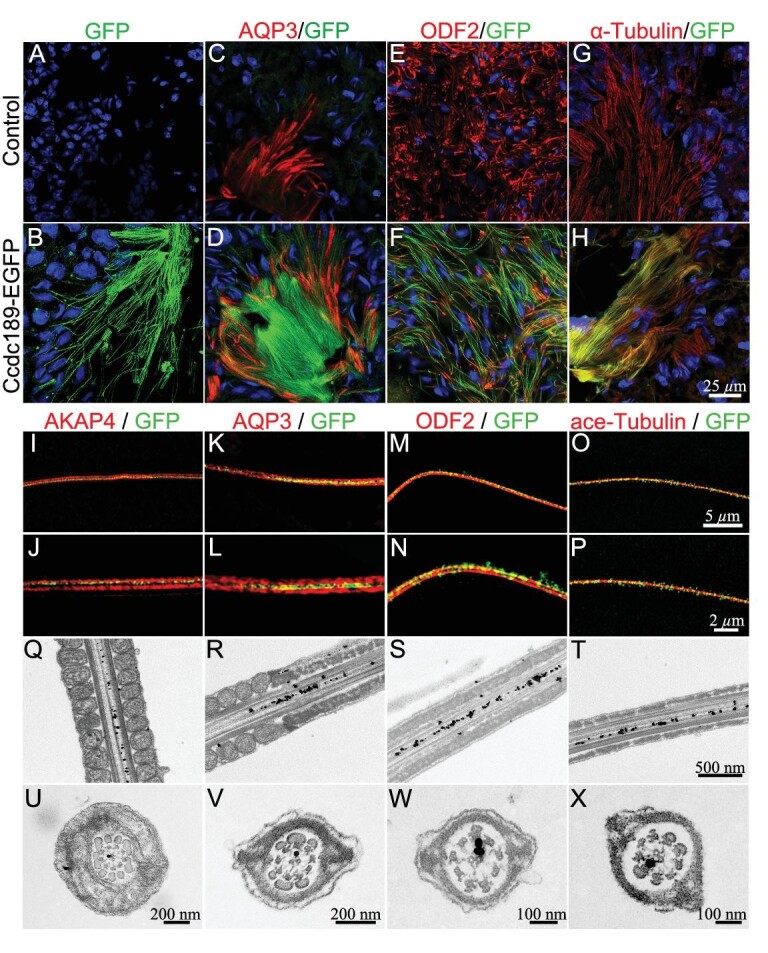
CCDC189 is located at the radial spoke of the first microtubule doublet (MTD). (A–H) Co-immunofluorescence of GFP (green) with α-Tubulin (C and D, red), ODF2 (E and F, red) and AQP3 (G and H, red) in testes of control and *Ccdc189*-GFP mice. (I–P) High-resolution images of co-immunostaining of GFP (green) with ace-tubulin (I and J, red), ODF2 (K and L, red), AQP3 (M and N, red) and AKAP4 (O and P, red) in sperm tails. Sub-cellular location of CCDC189 in sperm was examined by immunoelectron microscopy with longitudinal section (Q–T) and cross section (U–X).

To determine the subcellular location of the CCDC189 protein in sperm, we used the CRISPR/Cas9 system to generate an EGFP-tagged *Ccdc189* knockin mouse model (Supplementary Fig. 6A). The mouse model was verified by PCR and western blot analysis (Supplementary Fig. 6B and C). To determine whether the functions of the CCDC189 protein are affected by fusion with the EGFP tag, we first assessed the fertility of homozygous *Ccdc189*-EGFP mice and found that homozygous *Ccdc189*-EGFP male mice were fertile (Supplementary Fig. 6D–F). The process of spermatogenesis was normal, and a large number of mature sperm were observed in the epididymis of the homozygous *Ccdc189*-EGFP mice, and a *Ccdc189*-EGFP tagged allele could rescue the fertility defect of a *Ccdc189* knockout allele in the *Ccdc189^−/^*^EGFP^ mice, supporting the idea that EGFP-tagged protein is similar to wild-type *Ccdc189* in function (data not shown). These results indicated that the EGFP tag did not affect the functions of the CCDC189 protein. The immunostaining results showed GFP-positive fiber-like sperm flagella in *Ccdc189-*GFP mice, but not in control mice. GFP co-localized with AQP3, ODF2 and α-tubulin in testes, indicating that the *Ccdc189* protein was mainly located in sperm flagella (Fig. 3A–H).

To further determine the subcellular location of CCDC189 in sperm flagella, immunostaining was performed to detect GFP, AQP3, AKAP4, ODF2 and ace-tubulin in sperm, and high-resolution images were obtained (Fig. 3I–P). The sperm tail membrane was labeled with AQP3 (Fig. 3K and L), and the fibrous sheath of sperm tails was labeled with AKAP4 (Fig. 3I and J). We found that the GFP signal was located inside the AQP3-positive tail membrane (Fig. 3K and L) and AKAP4-positive fibrous sheath (Fig. 3I and J). We also used ODF2 to label the outer dense fiber (Fig. 3M and N) and ace-tubulin to label the microtubule structure (Fig. 3O and P). The results showed that the scattered GFP signal co-localized with the ace-tubulin-positive microtubule structure (Fig. 3O and P). These results indicated that *Ccdc189* was localized to the vicinity of the microtubule structure inside the flagellum of a spermatozoon.

To further determine the subcellular location of the *Ccdc189* protein at the ultrastructural level, immunoelectron microscopy was performed. As shown in Fig. 3, immunogold-labeled particles were detected between the microtubules of the middle piece (Fig. 3Q), principal piece (Fig. 3R and S) and tail end (Fig. 3T), as prepared by longitudinal sectioning. The golden particles were all located on one side of the central axis, indicating the polarized location of CCDC189 in the axoneme of the flagellum. To determine whether CCDC189 displays a regular polarized localization pattern in the flagellum, we further examined the location of golden particles in cross sections of sperm tails. As shown in Fig. 3U–X, the golden particles were located at the radial spoke of the first peripheral microtubule doublet. This result confirmed the polarized location of CCDC189 in sperm flagella.

### CCDC189 interacts with CABCOCO1 in the flagellum of spermatids

**Figure 4.**
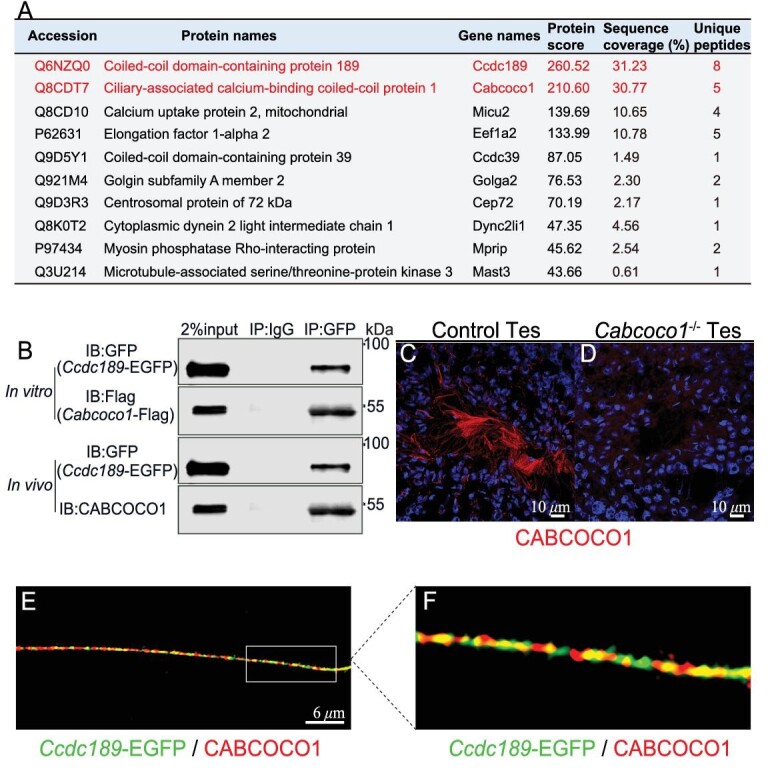
CABCOCO1 interacts with CCDC189. (A) List of the top 10 proteins which were pulled down by CCDC189 in testes. (B) The interaction between CCDC189 and CABCOCO1 was confirmed by Co-IP experiments *in vitro* and *in vivo*. (C) CABCOCO1 (red) was located in sperm flagellum in control testes. (D) No CABCOCO1 signal was detected in the testes of *Cabcoco1^−^^/^^−^* mice. (E and F) High-resolution images showed that CCDC189 (green) was partially co-localized with CABCOCO1 (red) in sperm flagellum.

To further explore the molecular mechanism of CCDC189 in sperm flagellum formation, CCDC189-interacting proteins in *Ccdc189*-EGFP mouse testes were identified by co-immunoprecipitation (co-IP) and mass spectrometry analyses, as shown in Fig. 4A and Supplementary Table 3. A total of 672 proteins in the testes of *Ccdc189*-EGFP mice were pulled down by an anti-GFP antibody, and 144 proteins were significantly enriched in *Ccdc189*-EGFP mice compared to wild-type control mice. We found that CCDC189 was the most highly enriched protein, in accordance with its protein-matching score. The 10 most highly enriched candidate CCDC189-interacting proteins are listed in Fig. 4A. The candidate proteins were verified via co-IP experiments. *In vitro* studies showed that the CABCOCO1 and CCDC189 proteins in HEK293 cells were mutually pulled down by overexpressed FLAG-tagged *Cabcoco1* and EGFP-tagged *Ccdc189* (Fig. 4B, top panel). The CABCOCO1 protein in the testes of *Ccdc189*-EGFP mice was also pulled down by an anti-GFP antibody (Fig. 4B, bottom panel). These results indicated that CABCOCO1 interacted with the CCDC189 protein in testes. The location of CABCOCO1 in sperm flagella was evaluated via immunofluorescence assay (Fig. 4C and D). High-resolution images showed that CCDC189 (green) was partially co-localized with CABCOCO1 (red) (Fig. 4E and F).

### Inactivation of the *Cabcoco1* gene causes aberrant sperm flagellogenesis

**Figure 5.**
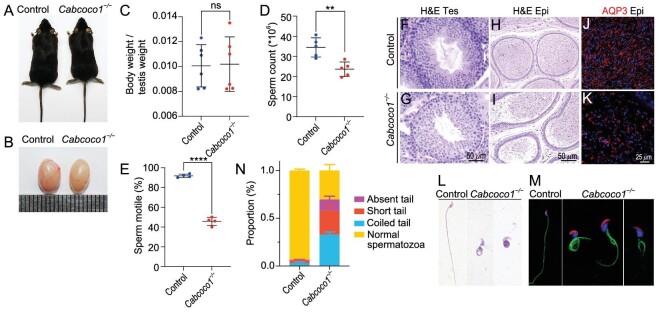
Inactivation of *Cabcoco1* causes defects in spermiogenesis. (A) No obvious developmental abnormalities were observed in adult *Cabcoco1*^−/−^ mice. (B) There was no difference in the size of testes in *Cabcoco1*^−/−^ mice compared to control mice. (C) The ratio of testis/body weight in *Cabcoco1*^−/−^ mice was not significantly changed. The (D) sperm count and (E) sperm motility were significantly reduced in *Cabcoco1*^−/−^ mice compared to control mice. The histology of the seminiferous tubules of (G) *Cabcoco1*^−/−^ mice was comparable to that of (F) control mice. A large number of mature sperm were observed in the epididymis of (H) control mice, and abnormal spermatozoa were observed in the epididymis of (I) *Cabcoco1*^−/−^ mice. Fiber-like AQP3 (red) positive sperm flagella were observed in the epididymis of (J) control mice, and a dot-like AQP3 (red) signal was detected in the epididymis of (K) *Cabcoco1*^−/−^ mice. The malformations of sperm flagella in *Cabcoco1*^−/−^ mice were examined by (L) HE staining and (M) immunofluorescence. (N) The percentage of sperm with abnormal flagella was significantly increased in *Cabcoco1*^−/−^ mice.

To identify the physiological functions of the *Cabcoco1* gene in spermatogenesis, we generated *Cabcoco1*-knockout mice using CRISPR−Cas9 technology. A null allele mouse model was generated by deleting all exons of the *Cabcoco1* gene (Supplementary Fig. 7A). The identity of the mouse model was verified by PCR (Supplementary Fig. 7B), western blotting (Supplementary Fig. 7C) and immunofluorescence staining (Fig. 4C and D). No obvious developmental abnormalities were observed in adult *Cabcoco1^−/−^* mice (Fig. 5A; Supplementary Fig. 7D), and the testes size of the *Cabcoco1^−/−^* mice was comparable to that of the control mice (Fig. 5B and C; Supplementary Fig. 7E). However, the sperm count and motility (Fig. 5D and E) were markedly reduced in the *Cabcoco1^−/−^* mice. No obvious histological defects were observed in the testes of the *Cabcoco1^−/−^* mice (Fig. 5F and G). However, a large number of abnormal spermatozoa were observed in the epididymis of the *Cabcoco1^−/−^* mice (Fig. 5H and I). AQP3-positive sperm flagella were observed in the epididymis and testes of the control mice (Fig. 5J; Supplementary Fig. 7F). In contrast, very few fiber-like AQP3 signals were observed in the epididymis and testes of the *Cabcoco1^−/−^* mice, and most of the AQP3 signal was punctate-like, which was consistent with that in the *Ccdc189^−/−^* mice (Fig. 5K; Supplementary Fig. 7G). The ODF2 and ace-tubulin signals were markedly reduced in the *Cabcoco1^−/−^*samples compared to the control samples (Supplementary Fig. 7H–K). The morphology of sperm from the caudal epididymis was further examined by H&E staining and immunofluorescence assay (Fig. 5L and M). A large proportion of abnormal sperm, including those with a missing tail, short tail or coiled tail, were observed in the *Cabcoco1^−/−^* mice, similar to the abnormalities observed in the *Ccdc189^−/−^* mice (Fig. 5N). These results indicated that *Cabcoco1* plays an important role in regulating the morphogenesis of sperm flagella.

### The interaction between CABCOCO1 and CCDC189 is mediated by the CCDC189 coiled-coil domain

**Figure 6.**
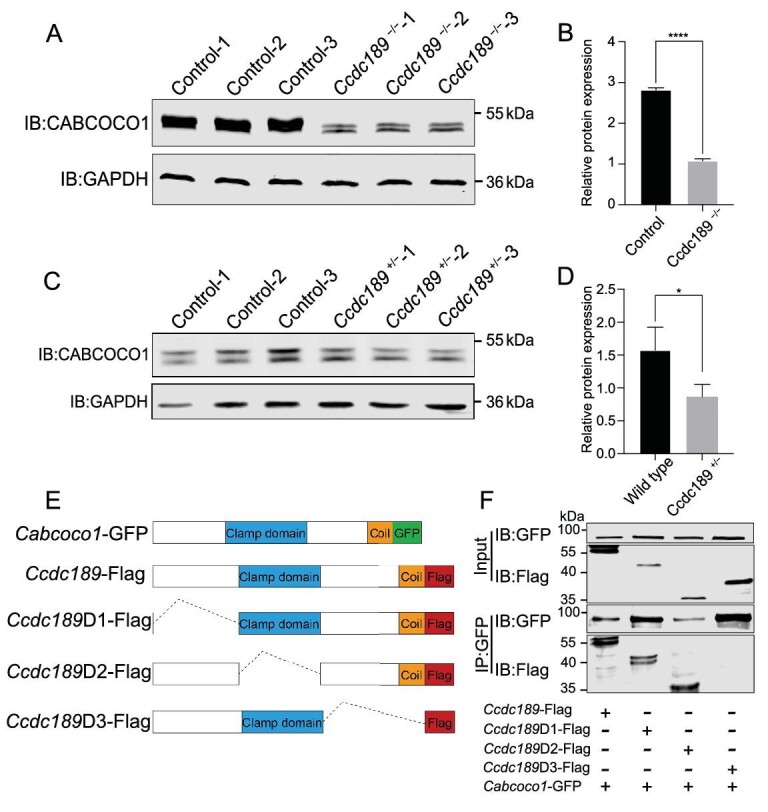
The interaction between CCDC189 and CABCOCO1 is mediated by the coil-coiled domain of CCDC189. The protein level of CABCOCO1 was significantly reduced in testes of both (A) *Ccdc189^−/−^* and (C) *Ccdc189^+/−^* mice. (B and D) Quantitative results of western blot analysis. (E) Schematic diagram of different truncated *Ccdc189* fragments. (F) D1 and D2 fragments of CCDC189 were pulled down by CABCOCO1.

To test whether the protein level of CABCOCO1 is affected in *Ccdc189*^−/−^ mice, the expression of CABCOCO1 was measured by western blotting. We found that the expression of CABCOCO1 was markedly reduced in *Ccdc189^−/−^* mice (Fig. 6A and B). We also found that the protein level of CABCOCO1 was significantly reduced in the testes of *Ccdc189^+/−^* mice (Fig. 6C and D). These results indicated that CCDC189 is required for the integrity maintenance of the CABCOCO1 protein and the inactivation of CCDC189 caused degradation of CABCOCO1. To further determine which domain is critical for the interaction between CCDC189 and CABCOCO1, a series of CCDC189 fragments were generated as indicated in Fig. 6E, and co-IP assays were performed by overexpressing the truncated proteins in HEK293 cells. As shown in Fig. 6F, we found that *Ccdc189*D3 (with the coiled-coil domain deleted) could not be pulled down by CABCOCO1, suggesting that the coiled-coil domain of CCDC189 is required for the interaction between these two proteins.

We also examined whether CCDC189 and CABCOCO1 interacted with other radial-spoke-associated proteins by performing co-IP experiments. As shown in Supplementary Fig. 8A and B, radial spoke head 1 homolog (RSPH1) was pulled down by both CABCOCO1 and CCDC189, indicating that CCDC189/CABCOCO1 complexes are involved in stabilizing the axoneme structure through their interaction with RSPH1. In addition to CABCOCO1, several intraflagellar transport (IFT)-related proteins, including IFT20 and IFT88, were also pulled down by CCDC189 as determined by IP/MS analysis. IFT20 and IFT88 are two major components of the intraflagellar transport machinery and form a module that is important for the flagellar assembly and maintenance [35,36]. To verify these results, a co-IP assay was performed. We found that the IFT88 protein in the testes of *Ccdc189*-EGFP mice was pulled down by an anti-GFP antibody (Supplementary Fig. 8C). We also found that both CCDC189 and CABCOCO1 interacted with IFT20 (Supplementary Fig. 8D). We also observed that IFT88 protein was diffused in germ cells and not co-localized with ace-tubulin in *Ccdc189-deficient* sperm (Fig. 2C–F). These results suggested that CCDC189 and CABCOCO1 are probably involved in intraflagellar transport during flagellum formation, as they interact with IFT-related proteins. Considering these results, we speculated that CCDC189 is probably required for intraflagellar transportation. When *Ccdc189* is inactivated, intraflagellar transport is negatively affected and the flagellar axoneme cannot assemble properly.

## DISCUSSION

Previous studies have demonstrated that CCDC family members play important roles in sperm flagellum formation, and mutations in these genes lead to MMAF in human patients and animal models [17]. Mutations in CCDC9 and CCDC151 are detected in patients with severe asthenozoospermia [37–39]. Biallelic mutations of CCDC39 and CCDC40 have been found in patients with ‘radial spoke defects’ in primary ciliary dyskinesia. Knockout of *Ccdc9, Ccdc38, Ccdc42, Ccdc62, Ccdc87* and *Ccdc136* results in male infertility in mouse models because of defects in sperm flagella [31,39–43]. *Ccdc189* also belongs to the CCDC family and has not been previously reported to be associated with MMAF in human patients [17]. In this study, we found that the inactivation of *Ccdc189* in a mouse model caused male infertility with malformation of sperm flagella, indicating that the CCDC189 protein also plays a critical role in sperm flagellum formation.

Sperm motility is driven by motile cytoskeletal elements in the tail called axonemes. The axoneme is composed of 9 + 2 microtubules, where a central pair of microtubules is surrounded by nine peripheral MTDs in a specific order [44]. The MTD that is perpendicular to the central pair of microtubules is defined as the first MTD, and MTDs 2–9 are named in clockwise order [45]. Radial spokes are T-shaped structures that link peripheral microtubule doublets to the central pair of microtubules. The spoke stalk binds to the A-tubule of each MTD, and the spoke head faces towards the center of the axoneme [46,47]. Radial spokes are prominent structural features of 9 + 2 axonemes and are crucial for flagellar beating. Each spoke consists of a thin stalk and a bulbous head [48]. The spoke heads contain structural proteins that specialize in having contact with the central apparatus, while the stalk contains regulatory elements for modulating flagellar beating. Previous studies have found that mutations in RSPH1 and CFAP61 cause disintegration of the sperm flagellar structure and primary ciliary dyskinesia [23,49]. In this study, we found that the CCDC189 protein was specifically located at the radial spoke. Most interestingly, the CCDC189 protein was located only at the radial spoke of the first MTD. The MTDs of sperm tail axonemes are defined from 1 to 9 in clockwise order. Whether these nine MTDs are functionally different is unknown. It has been reported that mutation of DNAH17 leads to the destabilization of MTDs 4–7 in both human patients and mouse models [50]. Frequent loss of MTD 7 has also been observed in *Ttll9-deficient* mice [51]. VDAC3-deficient mice are infertile with abnormal axonemes and the missing doublets correspond to the last four doublets [52]. In this study, we found that the CCDC189 protein was specifically located at the radial spoke of the first MTD by immunoelectron microscopy. Taken together, these results suggest that the MTDs of sperm axonemes are most likely functionally different and that the composition of different radial spokes is probably also different. However, the exact functions of different MTDs need to be further investigated.

CABCOCO1 contains a CLAMP domain in the center and a coiled-coil domain in the C-terminal region. A previous study found that CABCOCO1 is located at the centrosome of spermatocytes and round spermatids. In mature sperm, CABCOCO1 is located in the tail flagella [53]. However, the functions of *Cabcoco1* in sperm development have not been investigated previously. In this study, we found that CABCOCO1 interacted with CCDC189. Importantly, *Cabcoco1*^−/−^ mice also exhibited abnormal sperm flagella, which was consistent with the findings in *Ccdc189*^−/−^ mice. However, the phenotypes observed in *Ccdc189*^−/−^ mice were much more severe than those in *Cabcoco1*^−/−^ mice, suggesting that *Ccdc189* probably plays a more important role in flagellum formation. We also found that the protein level of CABCOCO1 was significantly reduced in *Ccdc189*^−/−^ mice, suggesting that CCDC189 is required for integrity maintenance of the CABCOCO1 protein. RSPH1 is a major component of the radial spoke, and inactivation of this gene results in disintegration of the sperm flagellar structure and male infertility [49]. In this study, we found that both CCDC189 and CABCOCO1 interacted with RSPH1. These results confirmed that CCDC189 and CABCOCO1 are located at the radial spoke.

Two transport mechanisms are involved in sperm flagellogenesis: intramanchette transport (IMT) and IFT [54–56]. IFT is critical for normal sperm flagellogenesis, and IFT20 and IFT88 are two major components of the intraflagellar transport machinery [35,36].

In the present study, we found that IFT20 and IFT88 interacted with CCDC189 and that the IFT88 protein was scattered and not co-localized with ace-tubulin in *Ccdc189-deficient* sperm. These results suggest that CCDC189 may play an important role in flagellogenesis through its facilitation of intraflagellar transportation. However, the exact function of CCDC189 in sperm flagellar morphogenesis needs further investigation.

**Figure 7.**
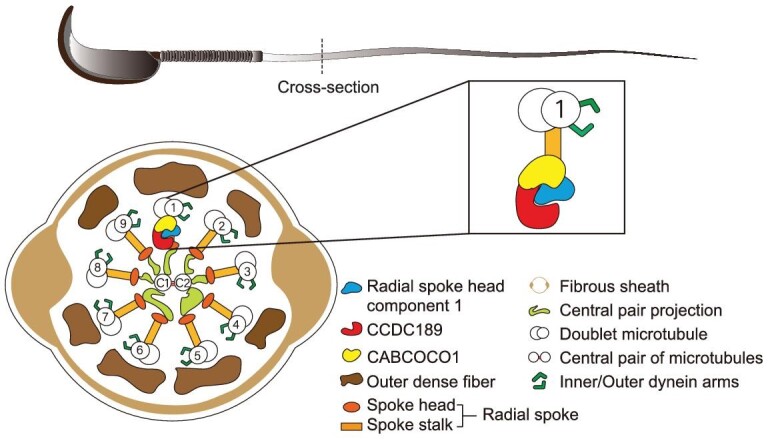
Schematic model of the functions of CCDC189 in sperm flagella formation. CCDC189 is involved in sperm flagella assembly or structure maintenance; it interacts with CABCOCO1 and other radial spoke proteins.

In summary, our study demonstrated that *Ccdc189* is a radial-spoke-associated protein and plays an essential role in sperm flagella formation (Fig. 7). *Ccdc189*-knockout mice displayed typical MMAF with aberrant sperm flagella. We discovered a new flagellar protein, CABCOCO1, which interacts with CCDC189 and RSPH1. Inactivation of *Cabcoco1* also led to flawed sperm flagella formation in mice. The similar symptoms imply that CCDC189 and CABCOCO1 are essential for male mouse spermatogenesis and fertility and may have similar functions in human spermatogenesis. Our research also revealed that different radial spokes in the MTDs of sperm axonemes are likely functionally diverse and composed differently. Our findings provide insights into the molecular cause of male infertility, and offer potential targets for developing new therapeutic strategies.

## MATERIALS AND METHODS: ANIMALS

All animal experiments were carried out according to the protocols approved by the Institutional Animal Care and Use Committee (IACUC) of the Institute of Zoology, Chinese Academy of Sciences (CAS; SYXK 2018-0017). All mice were maintained on a C57BL/6;129/SvEv mixed background and housed in specific pathogen-free (SPF) conditions under a 12 h light–dark cycle with a room temperature of 20–24°C and humidity of 35 ± 4%. *Ccdc189*-knockout mice were generated using the CRISPR-Cas9 system by Cyagen Biosciences. Cabcoco1- knockout mice and *Ccdc189*-EGFP knockin mice were generated using the CRISPR-Cas9 system by the Animal Experiment Center, Institute of Zoology. The primers used for genotyping are listed in Supplementary Table 1.

## Supplementary Material

nwad181_Supplemental_Files
