# Supplementary material for: CCDC189 affects sperm flagellum formation by interacting with CABCOCO1
Source: Natl Sci Rev. 2023 Jun 26;10(9):nwad181. doi: 10.1093/nsr/nwad181 (PMC10437088; doi:10.1093/nsr/nwad181)
Supplement: nwad181_Supplemental_Files [file nwad181_supplemental_files.zip › Supplementary figures.docx]

**Supplementary figures**


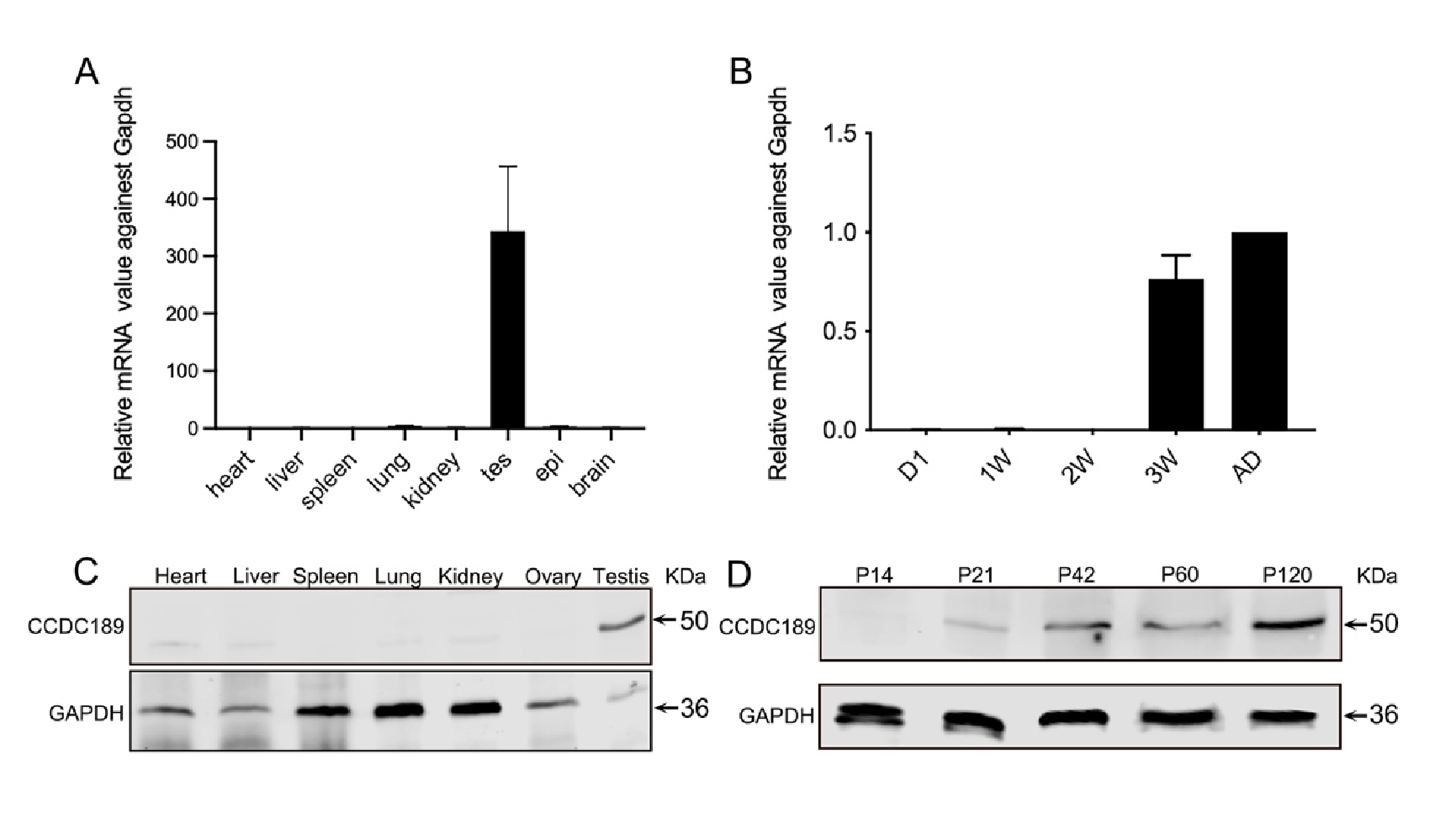


**Figure. S1. CCDC189 is specifically expressed in testis.**

A. High level of *Ccdc189* mRNA was detected in testes by RT-qPCR analysis. B. The mRNA level of *Ccdc189* was dramatically increased from 3 week in testes. C. High level of CCDC189 protein was detected in testes by Western blotting analysis. D. CCDC189 protein was detected in testes from D21 by Western blotting analysis.


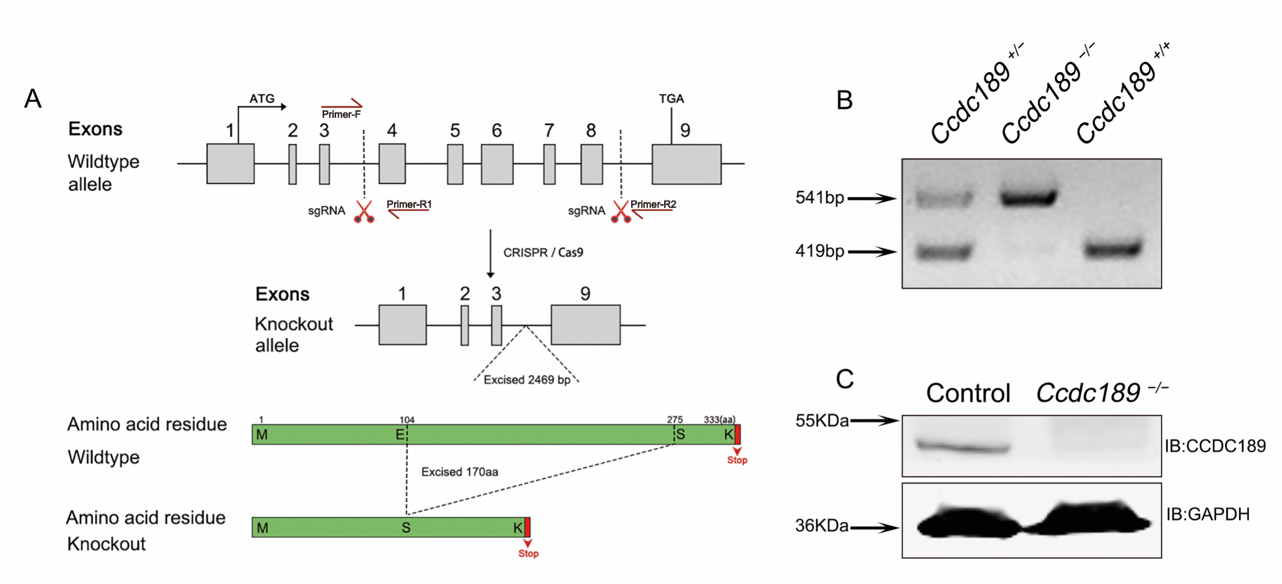


**Figure. S2. Generation of *Ccdc189* knockout mouse model.**

A. Schematic diagram showed the strategy for generating *Ccdc189* knockout mouse model. Exon 4 to 9 of *Ccdc189* gene was deleted using CRISPR-Cas9 system. Red arrows indicate the position of primer binding sites for genotyping analyses. B. Genotyping of *Ccdc189* knockout mice. C. Immunoblotting of CCDC189 in Control and *Ccdc189^−/−^* testes.


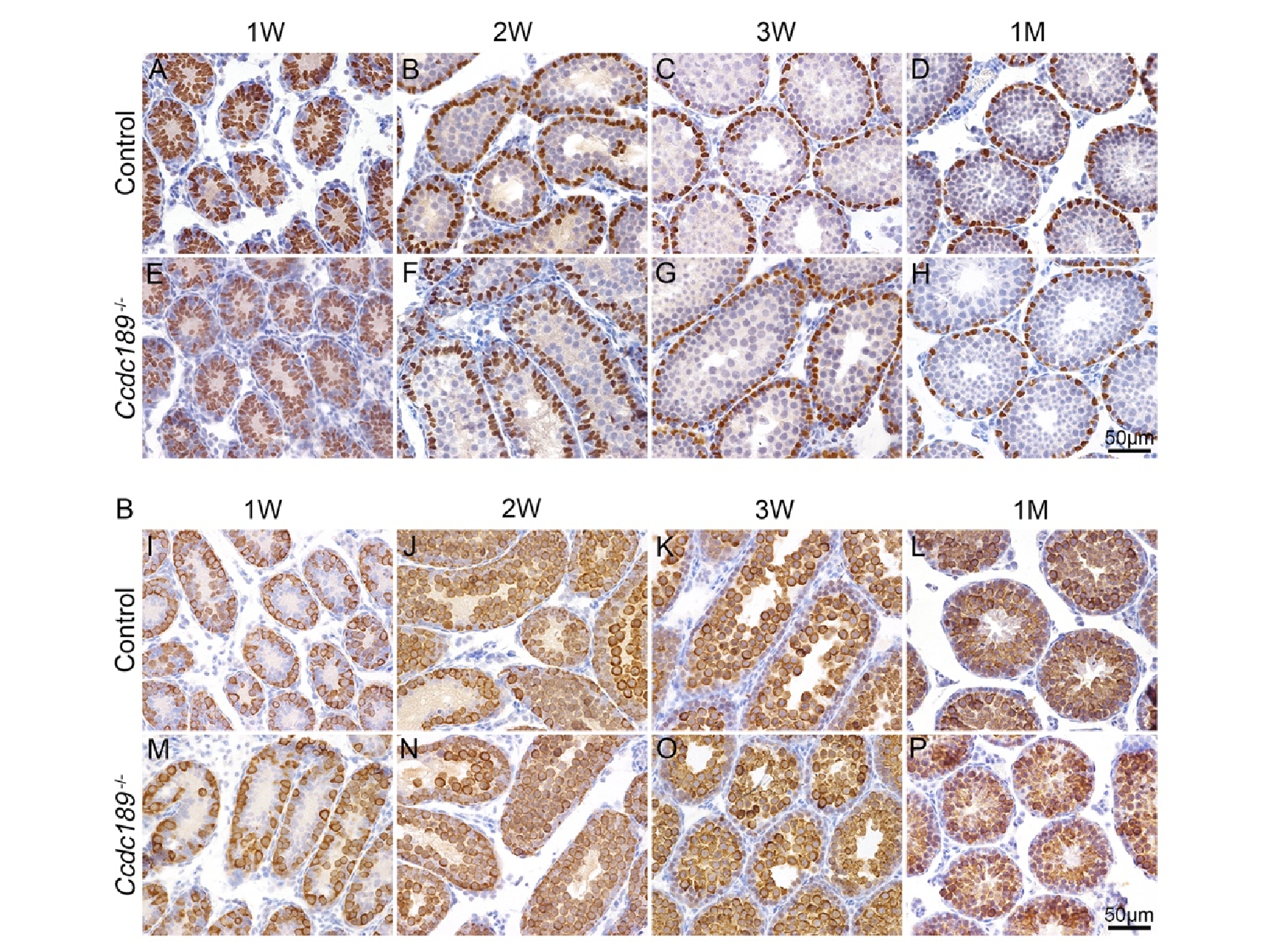


**Figure. S3. The early stage of germ cell development is not affected in *Ccdc189^−/−^* mice.**

A-H. The expression of Sertoli cell specific marker gene Sox9 (brown) in control and *Ccdc189^−/−^* mice was examined by immunohistochemistry. I-P. The expression of germ cell specific marker gene Ddx4 (brown) in control and *Ccdc189^−/−^* mice was examined by immunohistochemistry.


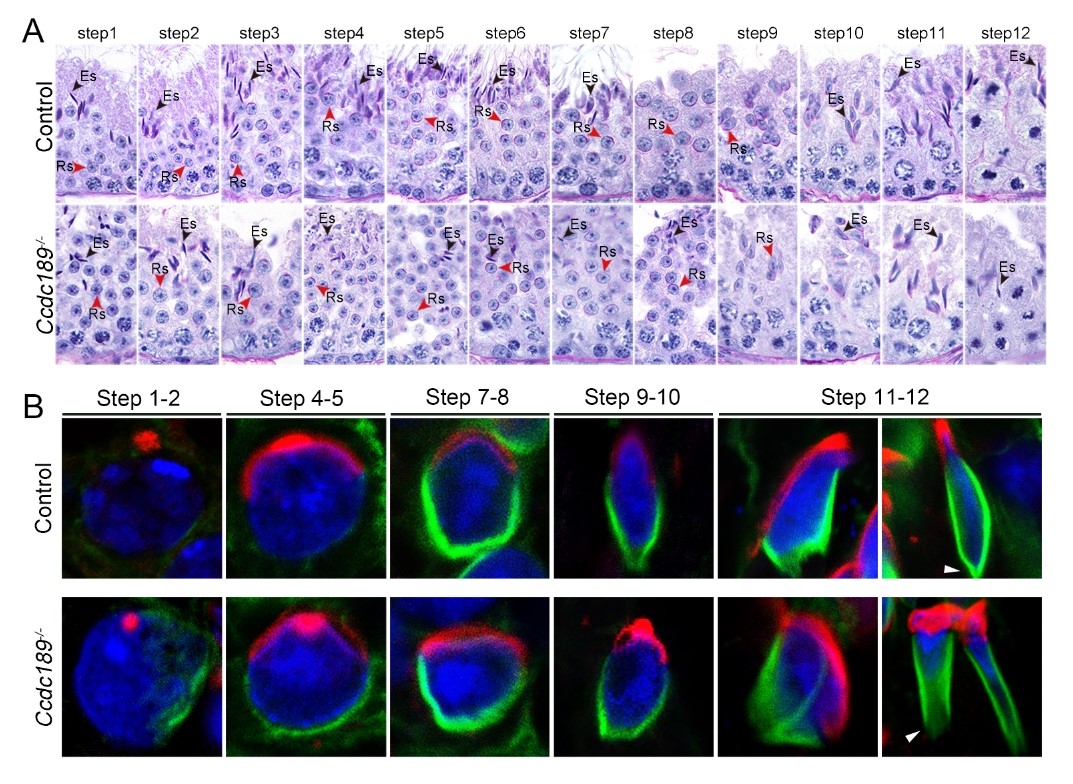


**Figure.S4. Abnormal manchette elongation is observed in *Ccdc189*^−/−^ mice during spermatogenesis.**

A. PAS-hematoxylin staining of control and *Ccdc189*^−/−^ seminiferous tubules at 8 weeks of age. No obvious abnormalities were observed in all 12 steps of seminiferous tubules epithelial cycle of *Ccdc189*^−/−^ mice. B. Immunofluorescence showed the abnormal extension of the manchette in elongating spermatids in Step11-12 of *Ccdc189*^−/−^ mice. The manchette was stained with α-tubulin (green), the acrosome was stained with PNA lectin histochemistry (red), and the nucleus was stained with DAPI (blue). Rs: round spermatids; ES: elongated spermatids.


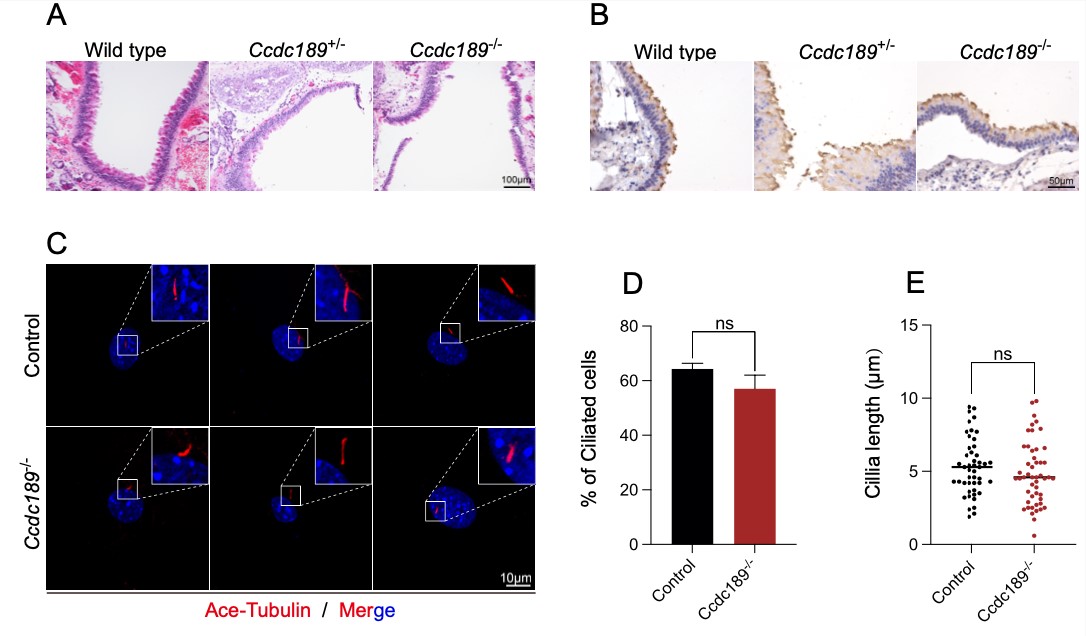


**Figure. S5. No defect in airway and starvation-induced ciliogenesis are noted in *Ccdc189* mice.**

A. The histology of the tracheas in control, *Ccdc189*^+/−^, and *Ccdc189*^−/−^ mice was examined by H&E staining. B. The expression of cilia specific marker gene ace-tubulin (brown) in control and *Ccdc189*^+/−^, *Ccdc189*^−/−^ mouse tracheas was examined by immunohistochemistry. C. Immunofluorescence of serum starvation induced ciliation in control and *Ccdc189*^−/−^ MEFs. The primary cilia (red) was stained with anti-acetylated tubulin antibodies. D-E. Quantification of ciliation and cilia length in MEFs.


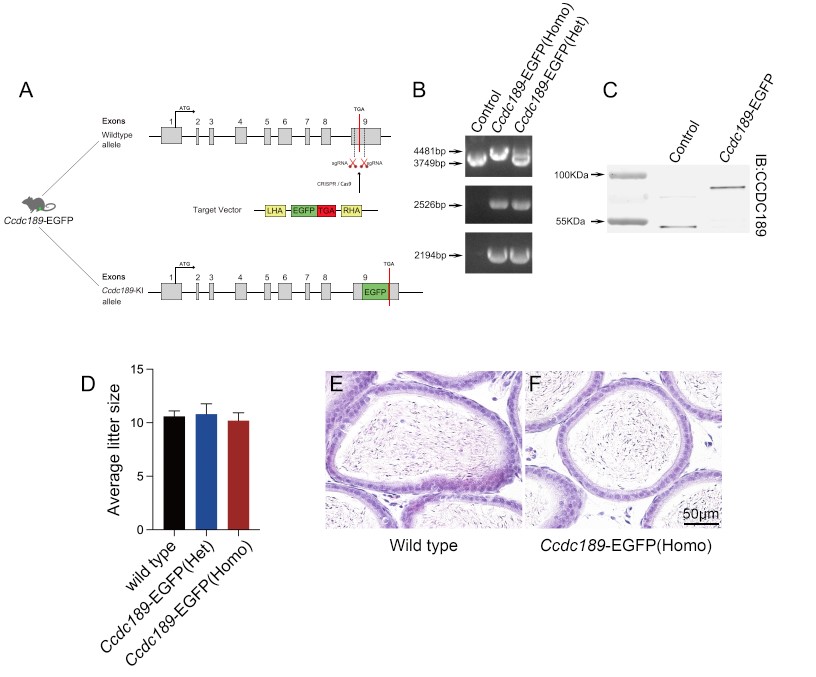


**Figure. S6. Generation of *Ccdc189-EGFP* knock-in mouse model.**

A. Schematic diagram of strategy for generating EGFP-tagged *Ccdc189* knock-in mouse model using CRISPR/Cas9 system. LHA/RHA, left/right homologous arms. B. Genotyping of *Ccdc189-*EGFP knock-in mice. C. Immunoblotting of CCDC189-EGFP in Control and *Ccdc189-*EGFP testes. D. Fertility test of control and male *Ccdc189-*EGFP knock-in mouse. E-F. The histology of the testes and epididymis in *Ccdc189-*EGFP knock-in mouse and control mouse were examined by H&E staining.


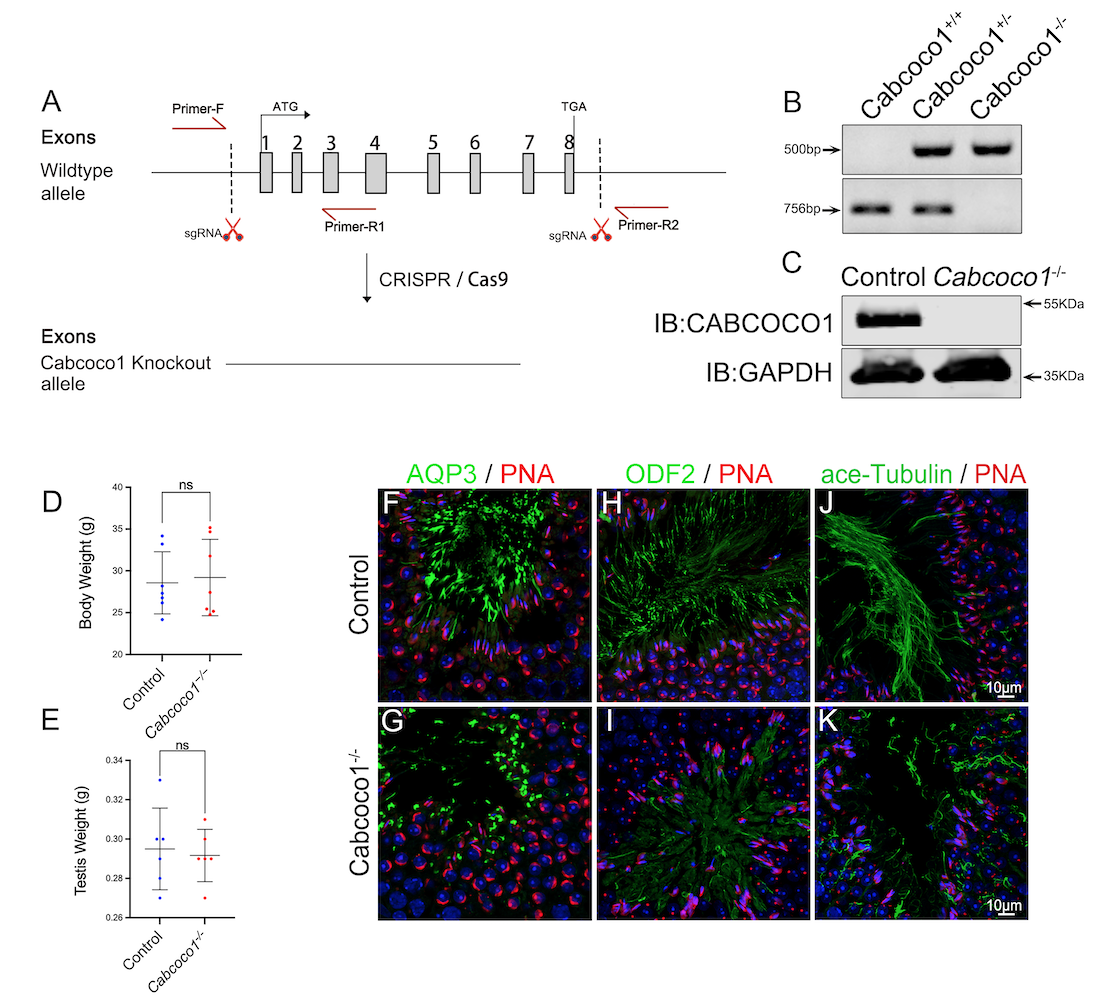


**Figure. S7. Generation of *Cabcoco1* knockout mouse model.**

A. Schematic diagram showed the strategy for generating *Cabcoco1* knockout mouse model. All exons of *Cabcoco1* gene were deleted using CRISPR-Cas9 system. Red arrows indicate the position of primer binding sites for genotyping analyses. B. Genotyping of *Cabcoco1* knockout mice. C. Immunoblotting of *Cabcoco1* in Control and *Cabcoco1^−/−^* testes. The body weight (D) and testis weight (E) of *Cabcoco1^−/−^* mice were not significantly changed. Immunofluorescence staining of AQP3 (green) and PNA (red) in testes from control and *Cabcoco1^−/−^* mice (F, G). ODF2(green) and acetylated tubulin (green) positive sperm flagella was observed in control testes (H, I) and very few acetylated tubulin (green) positive sperm flagella was detected in testes of *Cabcoco1*^−/−^ mice (J, K).


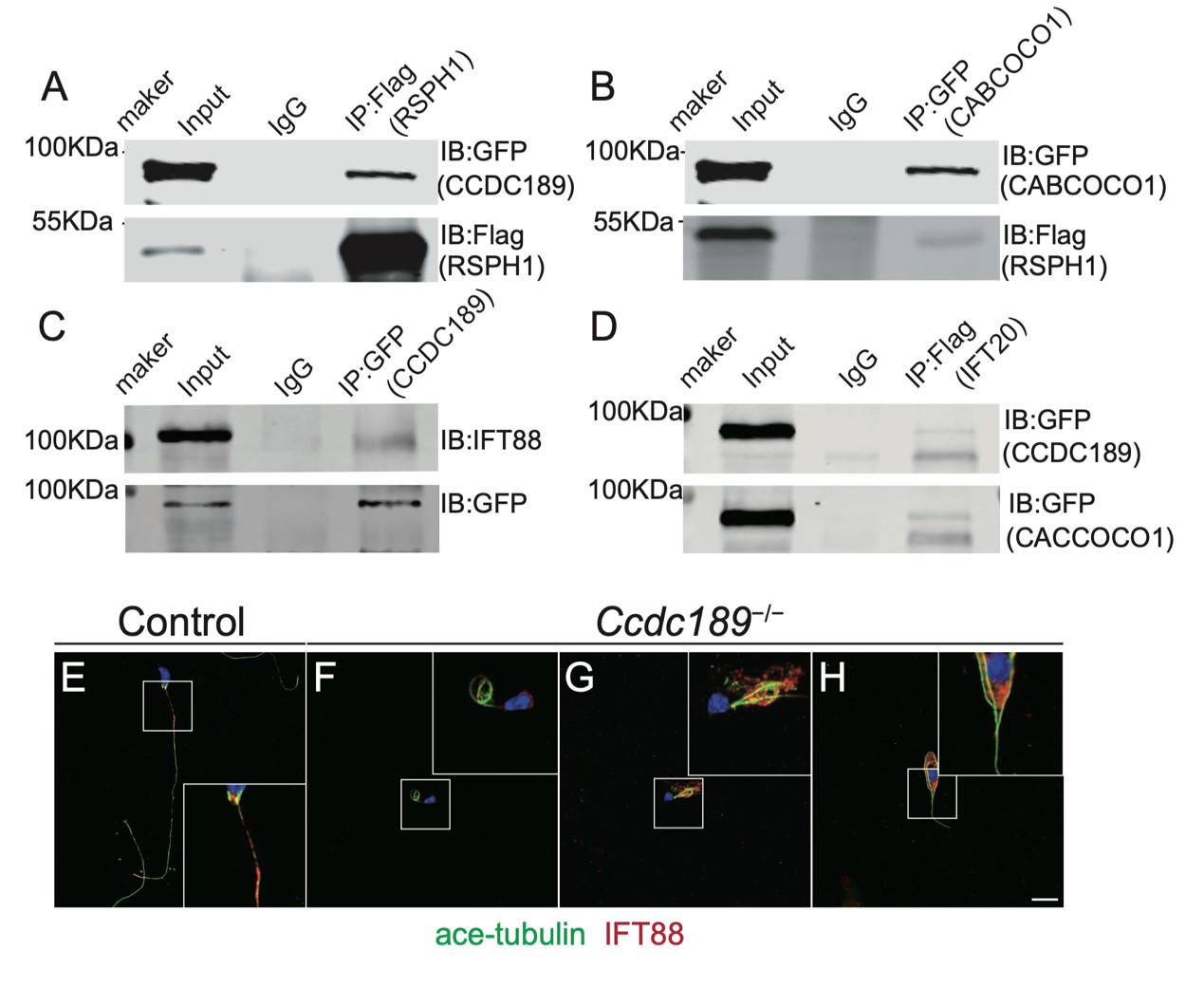


**Figure. S8. CCDC189 is interacted with RSPH1 and intra-flagellar transport (IFT) proteins.** HEK293T cells were transfected with pmcherry-Flag-*Rsph1*, pEGFP-GFP-*Ccdc189* (A), and pmcherry-Flag-*Rsph1*, pEGFP-GFP-*Cabcoco1* (B) expressing vectors. 48h after transfection, the cells were collected for immunoprecipitation (IP) with anti-Flag antibody and analysed with anti-Flag or anti-GFP antibodies. C. The interaction between CCDC189 and IFT88 was analyzed by co-IP experiments with *Ccdc189*-EGFP mouse testes. D. HEK293T cells were transfected with pRKFlag-Ift20, pEGFP-GFP-*Ccdc189* (Top row); pRK-Flag-*Ift20*, pEGFP-GFP-*Cabcoco1* expressing vectors (Bottom row). 48h after transfection, the cells were collected for immunoprecipitation (IP) with anti-Flag or anti-GFP antibodies and analysed with anti-Flag or anti-GFP antibodies.
