# Supplementary material for: CCDC189 affects sperm flagellum formation by interacting with CABCOCO1
Source: Natl Sci Rev. 2023 Jun 26;10(9):nwad181. doi: 10.1093/nsr/nwad181 (PMC10437088; doi:10.1093/nsr/nwad181)
Supplement: nwad181_Supplemental_Files [file nwad181_supplemental_files.zip › Supplemtnary Materials and methods.docx]

**Supplementary Materials and methods**

**RT-PCR**

Total RNA was extracted using RNeasy Kit (Aidlab, RN28) in accordance with the manufacturer’s instructions. 1 µg of total RNA was used to synthesize first-strand cDNA (Abm, G592). cDNAs were diluted and used for the template for real-time SYBR Green assay. Gapdh was used as an endogenous control. All gene expression was quantified relative to Gapdh expression.

The relative expression level of candidate genes was calculated using the formula 2-^ΔΔCT^. The primers for *Ccdc189* were summarized in supplementary table1.

**Histological analysis**

Tissues were fixed in 4% paraformaldehyde for up to 24 h, stored in 70% ethanol, and embedded in paraffin. Five-micrometer-thick sections were prepared and mounted on glass slides.

After deparaffinization, slides were stained with H&E for histologic analyses.

**Periodic acid–Schiff (PAS) staining**

PAS staining was performed as previously described (Sun and Handel, 2011)with modification. 5-µm paraffin sections of mouse testes were dewaxed and treated with 0.5% periodic acid for 30 min and Schiff’s reagent for 90 min. After being washed three times with distilled water, slides were then stained with hematoxylin and sealed with neutral gum.

**Immunohistochemistry (IHC) and Immunofluorescence (IF) Analysis**

Immunohistochemistry was performed as previously described (57). Paraffin sections of testes were incubated with the primary antibodies for 1 h. After washing with PBS, the slides were incubated with secondary antibodies. Slides were finally examined with confocal microscopy (Nikon, Japan), Images were captured by a Nikon DS- Ri1 CCD camera. The information for the antibodies was summarized in supplementary table2.

**High-resolution images**

The cauda epididymis was dissected from adult mice to obtain the sperm. The sperm were released from the cauda epididymis using PBS and incubated at 37℃ for 15 min. A volume of 10μl of the upper layer liquid was applied onto a glass slide and left to air dry naturally. Subsequently, the sample was fixed with 4% paraformaldehyde (PFA) for 5 min. The glass slide was then washed with PBS for 5 min, repeating this process three times. To prevent non-specific binding, the slide was blocked with 5% bovine serum albumin (BSA) at room temperature for one hour. The blocking solution was discarded, and the desired incubating antibody was diluted with 1% BSA. The slide was incubated at room temperature for 1 h. After the primary antibody incubation, the glass slide was washed with PBS for 5 min, repeating this process three times. Next, the secondary antibody was diluted with 1% BSA and incubated at room temperature for 1 h. Following the secondary antibody incubation, the glass slide was washed with PBS for 5 min, repeating this process three times. Finally, the slide was sealed using an anti-fluorescence quenching mounting medium. All high-resolution images were generated using the Elyra S.1 Zeiss Chimera system.

**Western Blotting**

Tissue and cell were washed with PBS, lysed with RIPA buffer (50 mM Tris–HCl [pH 7.5], 150 mM NaCl, 1% NP- 40, 0.1% SDS, 1% sodium deoxycholate, 5 mM EDTA) supplemented with protease inhibitors cocktail (Roche) and 1 mM PMSF. Equal amounts of total protein was electrophoresed using 10% SDS/PAGE gels. After electrophoresis, the proteins were transferred to a nitrocellulose membrane and probed with the primary antibodies. The images were captured with the ODYSSEY Sa Infrared Imaging System (LI-COR Biosciences, Lincoln, NE). Densitometry was performed using ImageJ software. The protein level was normalized to that of GAPDH.

**Immunoprecipitation**

Transfected HEK293T cells were lysed with IP buffer [50 mM Tris-HCl, 150 mM NaCl, 1% NP-40, 1mM EDTA, protease inhibitor cocktail (Roche)]. The extracted 1mg proteins were incubated with 1µg of primary antibodies overnight at 4◦C. Next, 50 μl of Protein A/G Magnetic Beads (MCE HY-K0202) were added to each incubation sample overnight at 4◦C. The beads were washed three times with 1×IP buffer. Finally, the immunoprecipitates were washed four times in lysis buffer supplemented with cocktail and PMSF, resolved in loading buffer, incubated for 5 min at 95 °C, and then analyzed by western blotting.

**Mass spectrometry analysis of pull down proteins**

To identify potential proteins that interact with CCDC189, we performed in vivo IP assay using *Ccdc189*-EGFP mouse testes lysis. Tissues were washed twice with phosphate-buffered saline (PBS)

and lysed in immunoprecipitation (IP) buffer (150mM NaCl, 1%NP-40, 5mM,

ethylenediaminetetraacetic acid (EDTA), 10 mM Tris–HCl [pH 7.5] ) , supplemented with Complete EDTA-free Protease Inhibitor Cocktail (Roche). Anti-GFP antibody (Abcam, ab6556) was added to a final concentration of 1 μg/ml lysate and incubated for 4h at 4°C. Lysates were then incubated with Protein A/G Magnetic Beads (MCE HY-K0202) for overnight at 4°C. After extensive washing with IP buffer for three times, the bound proteins were eluted with 30 μl of 2× sodium dodecyl sulphate (SDS) sample buffer. Samples were then separated by 4-15% SDS-page, followed by staining with Coomassie Blue (ThermoFisher, #24615). Then the gels peptides were identified by the LC-MS/MS analysis, which was performed by PTM BIO (Hangzhou, China).

**Epididymal sperm count**

The cauda epididymis was dissected from adult mice. Sperm were released from the cauda epididymis with PBS and incubated at 37℃ for 15 min. Then the sperm suspension was diluted at

1:100 and the sperm number were counted with a hemocytometer.

**Immuno-EM Analysis**

Pre-embedding immuno-electron microscopy analysis was performed as described previously (1) with a little modification. Sperm were harvested and fixed with 4% paraformaldehyde and 0.1% glutaraldehyde in 0.1M phosphate buffer (PB, pH 7.4) for 15 min at room temperature, and then storing in 2% PFA overnight at 4℃. After washing and quenching the free aldehyde with 50 mM glycine in PBS for 5 min, cells were permeabilized with 0.1% saponin in PBS for 30 min, blocked with 5% BSA (blocking solution) in PBS for 1 h. Then the cells were incubated with a primary antiGFP polyclonal antibody (1:300, Abcam, ab6556) diluted in blocking solution overnight at 4℃ and then a secondary anti-rabbit antibody (1.4 nm gold-conjugated Fab' fragment diluted 1:200, Nanoprobes, 2004). GoldEnhance™ EM Plus kit (Nanoprobes, 2114) was used to intensified the size of the 1.4 nm gold particles for 2 min. Following one wash with 1% sodium thiosulfate and several washes with Milli-Q water, samples were post-fixed with 1% OsO4 in 0.1 M PB for 30 min, stained with 1% aqueous uranyl acetate for 30 min, dehydrated with an ethanol series (30%, 50%,

70%, 85%, 95% and 2×100%, 5 min for each), infiltrated and embedded in EMbed 812 resin (Electron Microscopy Sciences, #14120). Resin was polymerized at 65 °C for 24 h. Consecutive plastic ultrathin sections (75 nm) were obtained with a diamond knife (ultra 35°, Diatome, Switzerland) using an ultramicrotome (EM UC7, Leica Microsystem), and mounted on formvar film-coated copper grids with a single slot, counterstained with uranyl acetate and lead citrate by standard methods. Stained sections were analyzed at 120kV on a Tecnai G2 Spirit BioTWIN transmission electron microscope (FEI) and photographed with a digital camera (Orius 832, Gatan). **Transmission Electron Microscopy**

The tissues were fixed with 2.5% glutaraldehyde in 0.1 M phosphate buffer (pH 7.4) overnight at 4°C. After being washed five times in phosphate buffer at 4°C, the testes were fixed in 1% OsO4 incubated on the ice for 1h and then washed five times with ddH2O. Then, the tissues were dehydrated in graded acetone (30%, 50%, 70%, 80%, 90%, 95%) for 5 min each step and finally in pure acetone two times for 10 min each. The tissues were infiltrated with SPURR resin. Ultrathin sections were cut with a Leica ultramicrotome. After serial staining with uranyl acetate and lead citrate, the tissues were photographed with 80 kV transmission electron microscope (JEM-1400,

JEOL, Japan).

**Scanning Electron Microscope**

Epididymal tissue was dissected and quickly cut up in 1 ml of PBS (to be used after filtration). After 10 min of release at 37°C, the released sperm were collected. Washed with PBS and spined down at 500g for 5 min. The sperm sediment was resuspended in 2.5% glutaraldehyde solution and fixed overnight at 4 °C. The sperm sediment was removed by centrifugation at 500 g for 5 min, and the residue was washed several times with 1 ml of 0.1 M PB. The resuspended sperm was then transferred to a paper packet made of filter paper and dehydrated in a gradient of alcohol (75%, 85%, 95%) for 15 min each. Finally, the paper packet was placed in 100% ethanol and soaked for 15 min. This procedure was repeated three times. The paper packets were dried at the critical point, and the sperm was then adhered to a metal table and sprayed with gold. After gold spraying, the samples were ready for scanning electron microscopy observation and imaging.

**Statistical analysis**

All experiments were repeated at least three times. 3–5 mice for each genotype at each time point were used for immunostaining or quantitative experiments. For immunostaining, one representative picture of similar results from 3 to 5 mice for each genotype at each time point was presented. The quantitative results were presented as the mean ± SEM. Statistical analyses were conducted using GraphPad Prism version 9.0.0. Unpaired two-tailed Student’s t-tests were used for comparison between the two groups. For three or more groups, data were analyzed using one-way

ANOVA. p-Values<0.05 were considered to indicate significance.

1. Chai, PY, Cheng, YR, Hou, CY, *et al.* USP19 promotes hypoxia-induced mitochondrial division via FUNDC1 at ER-mitochondria contact sites. *Journal of Cell Biology.* 2021; 220(7).
